# Characterization of rock joint surface anisotropy considering the contribution ratios of undulations in different directions

**DOI:** 10.1038/s41598-020-74229-z

**Published:** 2020-10-13

**Authors:** Man Huang, Chenjie Hong, Chengrong Ma, Zhanyou Luo, Shigui Du

**Affiliations:** 1 Department of Civil Engineering, Shaoxing University, 508 Huancheng West Road, Shaoxing, 312000 Zhejiang China; 2 Geotechnical Engineering Institute, Zhejiang University of Science and Technology, 318 Liuhe Road, Hangzhou, 310023 Zhejiang China

**Keywords:** Geology, Petrology

## Abstract

Anisotropy in rock joint is strongly dependent on undulating surface morphology. Recent research of the morphology showed the parameter can express the different types of anisotropic characteristics of the joint surface separately. This report aims to analyze the common characteristic of the anisotropic distribution and exhibit the anisotropic variation trend. The joint morphology function consists of two morphology functions of regular plane in orthogonal directions, and the anisotropic variation determined by the contribution ratios of the two morphology. The roughness weight ratio in orthogonal direction of joint surface is used as an index to describe the anisotropic variation behavior, which proposes the anisotropic variation coefficient (*AVC*). On this basis, it is divided into 5 levels from strong anisotropic to isotropic. According to the assumption of anisotropic arc distribution, the anisotropic analytic function is derived and the agreement between the deduced curves and measured data therefore suggests the possibility of defining the morphology anisotropy through the index *AVC.* Finally, we verify the characteristic of three natural rock joints, and prove the proposed function can reflect the anisotropic distribution trend. The new index can be used to describe the anisotropic variation behaviour of rock joint surfaces.

## Introduction

Affected by the complex geological formation movement, the discontinuities existing in rock mass will develop in different directions, causing the joint surface to become rough and anisotropic^1^. The existence of this particularity plays an important role in many deformation and failure phenomena of jointed rock mass^2–4^. There have been numerous experimental investigations on the anisotropic characteristics in rock joints, such as point load test, Brazilian test and shear test^5–8^. The studies show the surface roughness is considered as the most important factors influencing the anisotropic behaviour of rock joints^9–12^. However, due to the complicated distribution of surface morphology, the joint surface roughness is difficult to evaluate, which is actually the main reason for hindering the study of anisotropic mechanisms. Therefore, it is necessary to investigate the variation of morphology in rock joint surfaces with different directions.

The roughness parameterization of a joint surface provides a quantitative description of the anisotropy. According to the parameterization methods proposed by different scholars (see Supplementary Table S1 online), the parameters for describing the joint surface topography are mainly divided into two categories: directional and non-directional^13^. The former category analyzes the effective undulation of the potential contact portion of the surface. The latter one considers the overall fluctuation in the elevations of asperity. Both categories have been widely used as indicators in anisotropic description^14–20^. Though the studies show the roughness parameters can individually describe the anisotropic characteristics for the given joint surfaces, it cannot reflect the variation of the anisotropy with different surface roughness, and thus the study of anisotropy still staying at the property description stage. For so many anisotropic descriptions obtained in the previous studies, a common distribution law may exist that can parse the anisotropic behaviour, then the variation of anisotropy may be efficiently estimated. Therefore, a thorough investigation in the commonality of anisotropic characteristics between different joint surfaces needs to be carried out.

Quantitative characterization of anisotropy is part of safety assessments for rock engineering projects. Based on the study by Belem et al.^21–23^, the degree of anisotropy can be categorised by the ratio of the small half-axis and the large half-axis of the apparent anisotropy ellipse into five types: (i) surface morphology is anisotropic; (ii) surface is more anisotropic than isotropic; (iii) surface is more isotropic than anisotropic; (iv) surface morphology is considered as isotropic; (v) surface morphology is isotropic. The anisotropy is simplified to the ratio of the maximum directional roughness to the minimum directional roughness (2D or 3D) in any direction^13^. Unfortunately, this ratio has ignored the influence of surface morphology in other directions on the anisotropy. To clearly show the degree of anisotropy, the factor of the composition of joint surfaces should also be considered.

In this study, we propose a new method for constructing joint surfaces with contribution ratio based on the investigation of the anisotropic distribution in existing researches. Furthermore, a new index anisotropic variation coefficient (*AVC*) is defined for quantitatively describing the degree of variation in anisotropy. Moreover, according to the behaviour of anisotropy comprehensively described by the proposed method, an anisotropic analytic function is derived for analysing the variation of anisotropy. Finally, the proposed *AVC* and anisotropic analytic function are applied in natural rock joints, and the accuracy is proved. In doing so, the anisotropic variation behaviour of rock joint surfaces might be effectively assessed.

## Methodology

### Investigation of the anisotropy of rock joint surfaces

The anisotropy of a joint surface is illustrated by plotting the roughness parameters obtained in different directions on polar coordinates^24–27^. Theoretically, the shape of the anisotropy of the joint surface distributed on the polar diagram is a circle when the roughness parameters in all directions are the same, and the shape becomes an irregular arc when the roughness parameter changes to one or some directions. As shown in Supplementary Table S2 online, the distribution variation, which is controlled by the values of the roughness parameters and its direction, is clearly reflected in the anisotropic distribution maps. Although the selected roughness parameters and their dimension descriptions are different, there still is a specific variation trend, that all distribution features of anisotropy are basically orthogonal. In order to analyse this trend, we normalize the anisotropic distribution maps in Supplementary Table S2 online, and plot them in a polar diagram which have uniform orthogonal direction (see Supplementary Fig. S1 online). It is shown the distribution shape present "∞" when the roughness in ${9}0^{\circ}{-} {27}0^{ \circ }$ direction is zero. It means that the joint surface undulates only towards the $0^{ \circ }{-} {18}0^{ \circ }$ direction. To better distinguish these two orthogonal directions, the $0^{ \circ }{-} {18}0^{ \circ }$ direction with greater roughness is defined as the dominant direction, and the corresponding ${9}0^{ \circ }{-} {27}0^{ \circ }$ direction is the disadvantaged direction. when the roughness in disadvantaged direction gradually increases, the distribution shape like a "peanut shell" gradually expanded. Finally, the distribution changes to an "O" shape when the roughness is similar in both directions, and the joint surface is almost isotropic.

This phenomenon indicates that the complex joint surface morphology affected by geological movements in different directions may be explained by the superposition of two undulating surfaces in orthogonal directions, and the anisotropic characteristics can be approximatively determined by the morphological changes of the joint surface in these two directions. Therefore, we begin to study the variation characteristics of anisotropy by constructing the joint surface in orthogonal directions, which are helpful for determining the common of the anisotropic distribution in rock joints.

### Construction of anisotropic joint surface

**Figure 1:**
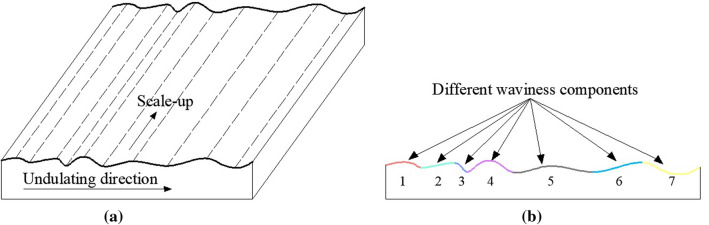
Simplified morphology of the joint surface. (**a**) Example of a regular rock surface; (**b**) morphology components of a joint profile.

To determine the common of the anisotropic distribution, we need to construct the rock joints with different undulating surfaces. Due to the complication of actual joint surfaces, it is necessary to consider the regular plane that undulating in one direction. As a special case in the study of joint surface, its morphology can be considered to be extended by the same profiles along the undulation direction (Fig. 1a). Thus, we first need to obtain the morphological function of a random profile. Yang et al. proposed the morphology of a joint profile can be approximated satisfactorily by using summation of several simple sine (or cosine) waves (Fig. 1b), and its morphological function *z(x)* varies periodically in x is defined with the magnitude *a*, the frequency *b* and the phase *c* as follows^28^:

1 $${\text{z}}\left( x \right) = \sum\limits_{i = 1}^{m} {a_{i} \sin \left( {2\pi b_{i} x_{i} + c_{i} } \right)}$$

**Figure 2:**
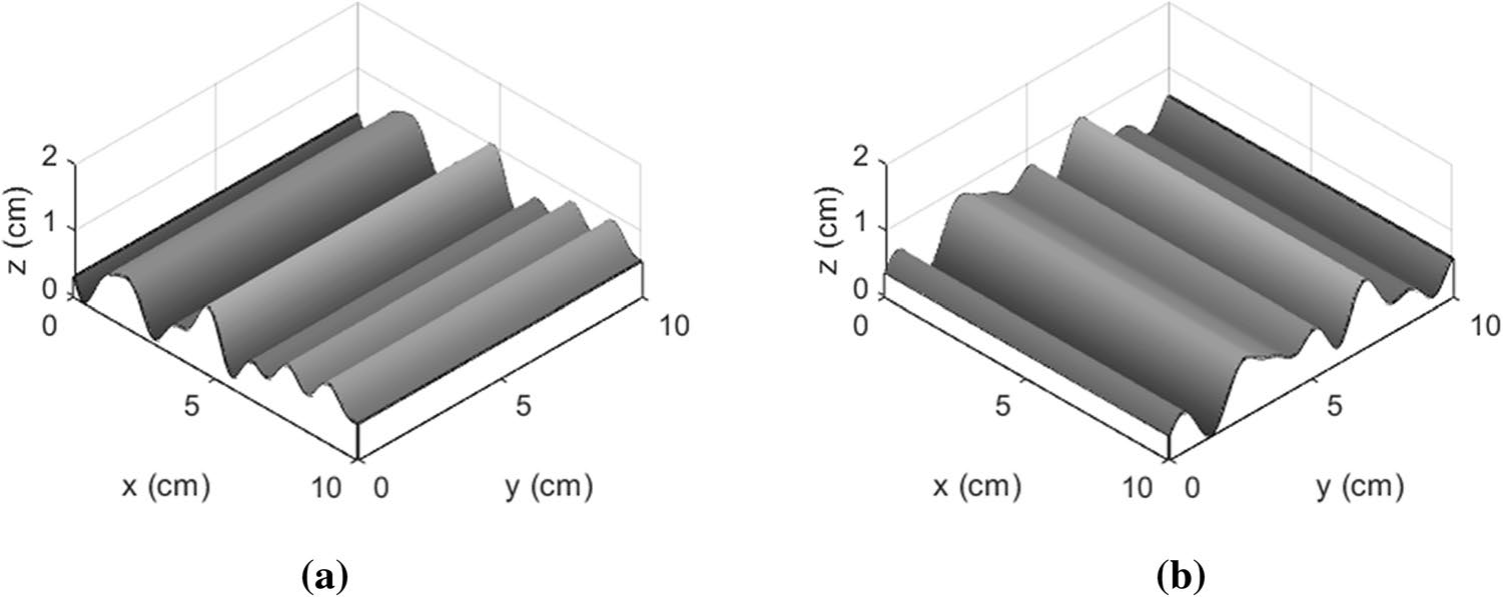
Three-dimensional undulating shapes of regular planes. (**a**) Undulations in the x direction; (**b**) undulations in the y direction.

Based on the above discussion, the three-dimensional description of joint surface is carried out by extending the two-dimensional morphological function of the x-axis direction in the coordinate system by *n* units along the y-axis, and its three-dimensional morphological function *Z(x)* is given by Eq. (2). Similar result of *Z(y)* is also obtained and given by Eq. (3). In Fig. 2, both images are plotted as a function of x and y.

2 $$Z\left( x \right) = \sum\limits_{i = 1}^{m} {a_{1ij} \sin \left( {2\pi b_{1ij} x_{ij} + c_{1ij} } \right)}$$

3 $$Z\left( y \right) = \sum\limits_{i = 1}^{m} {a_{2ji} \sin \left( {2\pi b_{2ji} y_{ji} + c_{2ji} } \right)}$$

**Figure 3:**
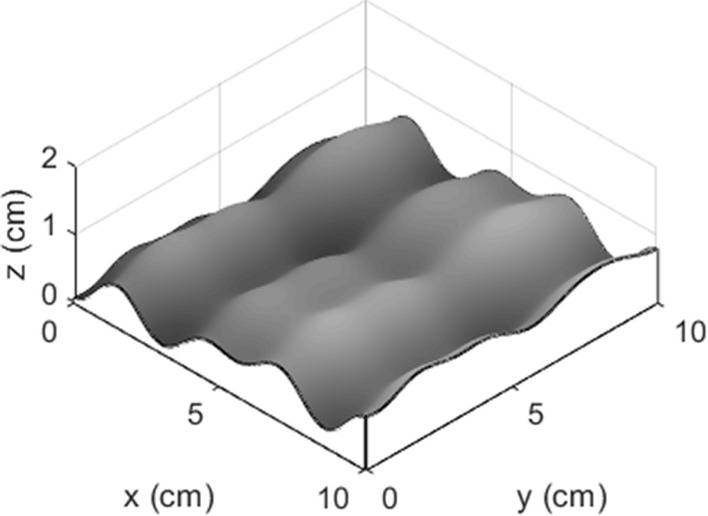
Superimposed morphology of an irregular plane (k1 = 0.7, k2 = 0.3).

The actual rock joint is not regular as the above surface morphology, and thus we cannot directly establish the function of spatial position. However, the commonality between these regular or irregular planes is their anisotropy. According to the analysis of the anisotropic distribution in “Methodology” section, the actual joint surface can be considered as formed by the superposition of the same regular planes in orthogonal directions, and its morphology depends on the contributions of these two directions to the undulation. Consequently, an irregular plane can be mathematically described by the two deterministic components of regular plane in orthogonal directions, and the morphological function is given by:

4 $$z = k_{1} \cdot Z_{x} + k_{2} \cdot Z_{y}$$

with $k_{1} + k_{2} = 1$. Here $Z_{x}$ and $Z_{y}$ represent the morphological functions of the regular plane in the x and y directions, respectively; $k_{1}$ and $k_{2}$ represent the contribution ratios of the x and y directions to the surface undulation, respectively. When $k_{1} = 1$ or $k_{{1}} = 0$, the joint surface exhibits a regular undulating morphology; when 0 < $k_{1}$ < 1, the joint surface exhibits an irregular undulating morphology (Fig. 3). Therefore, the joint surfaces with different undulating morphology can be characterized by the contribution ratios.

### Description of anisotropic variation behavior

To identify the anisotropic variation behaviour, the contribution ratio is first selected to characterize the joint surface with different undulating morphology. Then, by means of the roughness parameters, we describe the morphological characteristics in different orientations. Finally, the anisotropic distributions are drawn on polar coordinates. Considering that the $k_{1}$ has the same contribution relationship in the ranges of $\left[ {0,{0}{\text{.5}}} \right]$ and $\left[ {{0}{\text{.5}},{\kern 1pt} {\kern 1pt} {\kern 1pt} {1}} \right]$, except the undulating directions. Moreover, the x and y directions defined in this study are relative because the undulations of joint surfaces in these two directions are the same. Therefore, when $k_{1} \in \left[ {0,0.5} \right]$, it can fully characterize the degree of undulating contributions in different directions. In the process of selecting the roughness parameters, we determine to use the directional parameters to better reflect the directionality of the morphology. In addition, considering the influence of the 2D and 3D parameters on anisotropy, we choose ${{\theta_{\max }^{ * } } \mathord{\left/ {\vphantom {{\theta_{\max }^{ * } } {\left( {C + 1} \right)}}} \right. \kern-\nulldelimiterspace} {\left( {C + 1} \right)}}_{2D}$ and ${{\theta_{\max }^{ * } } \mathord{\left/ {\vphantom {{\theta_{\max }^{ * } } {\left( {C + 1} \right)}}} \right. \kern-\nulldelimiterspace} {\left( {C + 1} \right)}}$ to measure morphological characteristics for more comprehensively analysing the anisotropic variation behaviour of joint surfaces^29,30^. Their calculation are as follows:

5 $$L_{{\theta^{ * } }} = L_{0} \left( {\frac{{\theta_{\max }^{ * } - \theta^{ * } }}{{\theta_{\max }^{ * } }}} \right)^{C}$$

6 $$A_{{\theta^{ * } }} = A_{0} \left( {\frac{{\theta_{\max }^{ * } - \theta^{ * } }}{{\theta_{\max }^{ * } }}} \right)^{C} ,$$

where $L_{0}$ is the maximum potential contact length ratio, $A_{0}$ is the maximum potential contact area ratio, $\theta_{\max }^{*}$ is the maximum apparent dip angle in the shear direction, and *C* is the roughness fitting coefficient.

According to the proposed construction method, MATLAB is used to construct six different joint surfaces whose $k_{1}$ are 0, 0.1, 0.2, 0.3, 0.4 and 0.5 respectively, with each size being 100 mm × 100 mm and height variation being less than 10 mm. Then, by cropping the square sampling windows into circles, eighteen 2D profiles of the same nominal length are extracted at angular increments of 10° (see Supplementary Fig. S2 online), and the 2D parameter ${{\theta_{\max }^{ * } } \mathord{\left/ {\vphantom {{\theta_{\max }^{ * } } {\left( {C + 1} \right)}}} \right. \kern-\nulldelimiterspace} {\left( {C + 1} \right)}}_{2D}$ and 3D parameter ${{\theta_{\max }^{ * } } \mathord{\left/ {\vphantom {{\theta_{\max }^{ * } } {\left( {C + 1} \right)}}} \right. \kern-\nulldelimiterspace} {\left( {C + 1} \right)}}$ are used to measure the morphological characteristics. The anisotropic distribution from Table 1 shows that when the $k_{1}$ ∈ [0,0.5], the behaviours of anisotropy reflected by different directional parameters agree with the approximate results, in which the variations are all from anisotropic to isotropic. Among them, the anisotropic distribution reflected by 2D parameter has obvious fluctuation compared to 3D parameter. This is because the local geometry of the joint surface described by 2D parameter ignores the continuity of the morphology between different directions, which indicates that the anisotropy described by 3D parameter is more accurate.

**Table 1:**
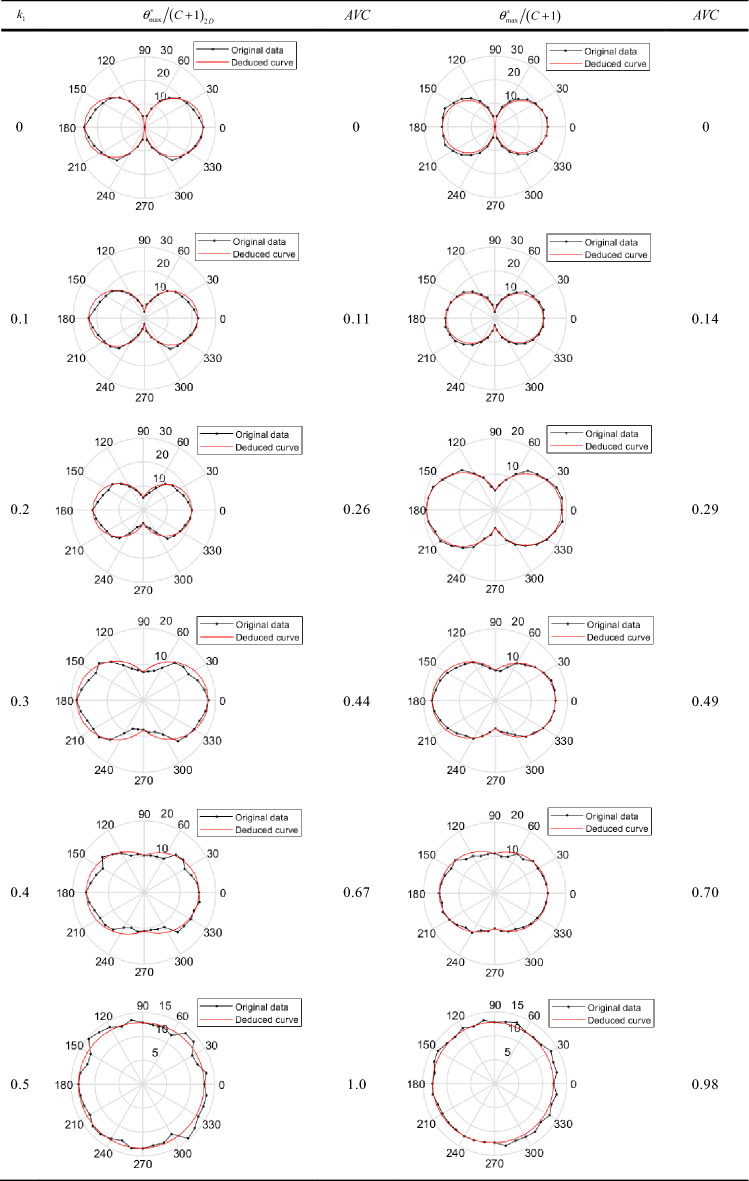
Anisotropy distribution of different contribution ratios.

## Analysis

### Index AVC for describing the variation of anisotropy

It is verified from the results in “Methodology” section that the anisotropic variation is determined by the joint morphology in orthogonal directions. Based on this, we define an index anisotropic variation coefficient (*AVC*), which quantifies the degree of anisotropic variation by calculating the weight ratio of roughness in the orthogonal direction. For one of the orthogonal directions, however, the joint surface has different roughness in its forward and reverse direction. Take the direction of $0^{ \circ }{-} 180^{ \circ }$ as an example, the roughness parameter in the forward direction ($0^{ \circ } \to 180^{ \circ }$) is not same as the reverse direction ($180^{ \circ } \to 0^{ \circ }$). So, the sum of those two roughness parameter values is taken as the weight of roughness to anisotropy in this direction. Then the *AVC* is determined as follows:

7 $$AVC{ = }\frac{{P_{{{90}}} + P_{{{270}}} }}{{P_{{0}} + P_{{{180}}} }},$$

where the $P_{{{90}}}$ and $P_{{{270}}}$ represent the forward and reverse roughness parameter in the direction of ${9}0^{ \circ }{-} {27}0^{ \circ }$, $P_{{0}}$ and $P_{{{180}}}$ represent the forward and reverse roughness parameter in the direction of $0^{ \circ }{-} 180^{ \circ }$.

According to the definition of *AVC*, we calculate the degree of anisotropic variation at different contribution ratios in Table 1. The results show that the smaller the *AVC* is, the more prominent the anisotropic variation behaviour is. To better quantify the degree of anisotropic variation, we refer to the anisotropic classification proposed by Belem et al.^21^ and provide a new anisotropic classification based on AVC which are shown in Table 2.

**Table 2:** Classification of anisotropic variation levels.

Range of the variation in *AVC*	Degree of anisotropic variation	
$0 \le AVC < 0.2{5}$	Anisotropic	Strong
$0.25 \le AVC < 0.{5}$		Moderately strong
$0.5 \le AVC < 0.{75}$		Medium
$0.75 \le AVC < 0.{9}$		Weak
$0.9 \le AVC \le 1$	Isotropic	

### Anisotropic analytic function

**Figure 4:**
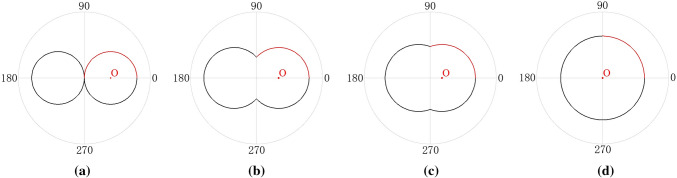
Analysis of anisotropic variation.

To explain the anisotropic variation, we assume that the values of roughness parameters in different directions fall on an arc. Therefore, the anisotropic distribution can be interpreted by the *AVC* as follows: When the *AVC* is close to 0, its distribution shape is approximately tangent to two circles, indicating that the joint surface undulates in only one direction (Fig. 4a); when the *AVC* in the range of (0,1), its distribution shape approximately changes to two intersecting circles. And the undulation of the vertical joint surface gradually increases as the *AVC* increases (Fig. 4b,c); when the *AVC* is close to 1, its distribution shape changes to a circular, indicating that the roughness parameters in each direction are similar (Fig. 4d). This phenomenon is closely related to the roughness parameters in the orthogonal directions. Therefore, we divide the anisotropic distribution into four parts and select arc segments with the range of (0, π/2) for anisotropic variation analysis (the red parts in Fig. 4).

According to the basic equation of the circular curve, we set *∆x* as the distance from the centre of the circle to the origin of the coordinates, the value of $P_{{0}}$ and $P_{{{90}}}$ are the polar radiuses which correspond to the polar angle *θ* are 0° and 90°, respectively (see Supplementary Fig. S3 online). Then, the roughness parameter P (ρ, θ) for any direction in the range of (0°, 90°) can be calculated by the following relationship:

8 $$\rho = \sqrt {P_{0}^{2} - 2P_{0} \cdot \Delta x + \left( {\Delta x \cdot \cos \theta } \right)^{2} } + \Delta x \cdot \cos \theta$$

9 $$\Delta x = \frac{{P_{0}^{2} - P_{90}^{2} }}{{2P_{0} }}$$

let $K_{{1}} = {{P_{90} } \mathord{\left/ {\vphantom {{P_{90} } {P_{0} }}} \right. \kern-\nulldelimiterspace} {P_{0} }}$, then, ∆x can be expressed as:

10 $$\Delta x = \frac{{P_{0} \left( {1 - K_{{1}}^{2} } \right)}}{2}$$

let $K_{\rho 1} = {{\left( {1 - K_{{1}}^{2} } \right)} \mathord{\left/ {\vphantom {{\left( {1 - K_{{1}}^{2} } \right)} 2}} \right. \kern-\nulldelimiterspace} 2}$ and substitute into Eq. (6), we obtain the following relationship:

11 $$\rho = P_{0} \left( {\sqrt {1 - 2K_{\rho 1} + \left( {K_{\rho 1} \cdot \cos \theta } \right)^{2} } + K_{\rho 1} \cdot \cos \theta } \right){\kern 1pt} {\kern 1pt} {\kern 1pt} {\kern 1pt} {\kern 1pt} {\kern 1pt} {\kern 1pt} {\kern 1pt} {\kern 1pt} {\kern 1pt} \theta \in \left( {0,{\pi \mathord{\left/ {\vphantom {\pi 2}} \right. \kern-\nulldelimiterspace} 2}} \right)$$

The above function explains the anisotropic variation of one arc segment in the range of $\left( {0,{\pi \mathord{\left/ {\vphantom {\pi 2}} \right. \kern-\nulldelimiterspace} 2}} \right)$. Hence, the anisotropic distribution function, which can be used to explain the entire anisotropic distribution, is obtained by calculating each arc segment and given by:

12 $$\rho = P \cdot \left( {\sqrt {1 - 2K_{\rho } + \left( {K_{\rho } \cdot \cos \theta } \right)^{2} } + K_{\rho } \cdot \left| {\cos \theta } \right|{\kern 1pt} } \right){\kern 1pt} {\kern 1pt} {\kern 1pt} {\kern 1pt} {\kern 1pt} {\kern 1pt} {\kern 1pt} {\kern 1pt} {\kern 1pt} \theta \in \left( {0,2\pi } \right)$$

where $P$ is the value of the roughness parameter in the direction of $0^{ \circ }{-} 180^{ \circ }$. When $\theta \in \left( {0,\pi {/2}} \right) \cup \left( {{{3\pi } \mathord{\left/ {\vphantom {{3\pi } {2,2\pi }}} \right. \kern-\nulldelimiterspace} {2,2\pi }}} \right)$, the value of $P$ is $P_{{0}}$. When $\theta \in \left( {{\pi \mathord{\left/ {\vphantom {\pi {2,{{3\pi } \mathord{\left/ {\vphantom {{3\pi } 2}} \right. \kern-\nulldelimiterspace} 2}}}} \right. \kern-\nulldelimiterspace} {2,{{3\pi } \mathord{\left/ {\vphantom {{3\pi } 2}} \right. \kern-\nulldelimiterspace} 2}}}} \right)$, the value of $P$ is $P_{{{180}}}$; ${\kern 1pt} K_{\rho }$ represents the calculated value related to the *K*_*1*_ in the corresponding angle range.

**Figure 5:**
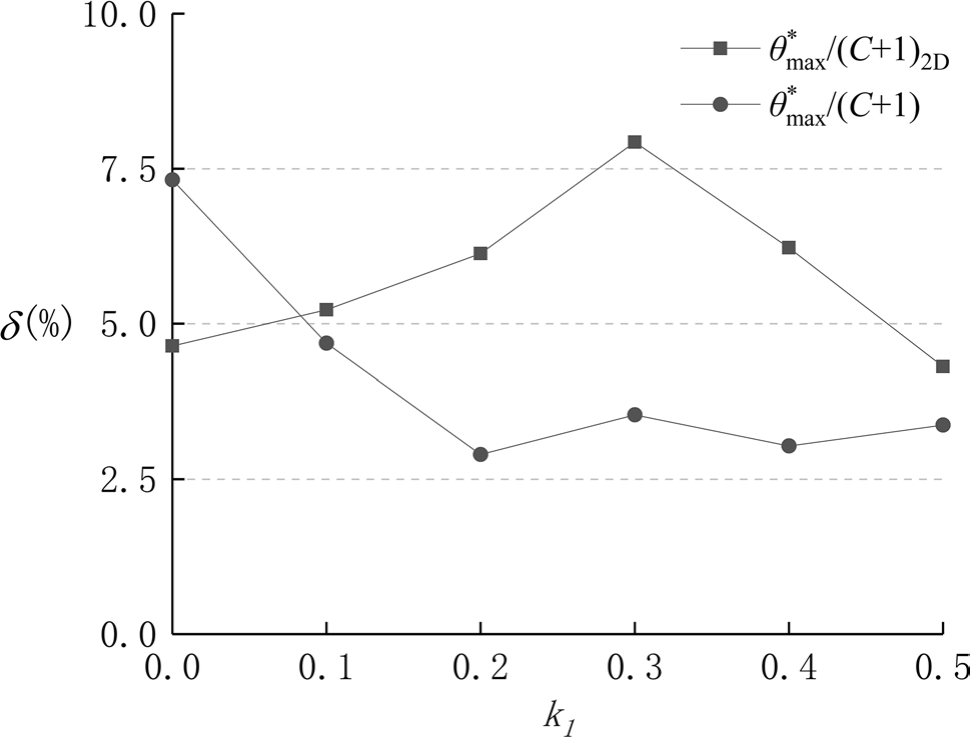
Error analysis of deduced curves and measured data.

Using the above function, the anisotropic reanalysis of the above six sets of joint surfaces with different contribution ratios is performed, and the anisotropic distributions are listed in Table 1 (the red curves). As a comparison of prediction effects, Fig. 5 gives an error analysis of the deduced curves and measured data, and the average relative error $\mathop \delta \limits^{ - }$ is calculated as follows:

13 $$\mathop \delta \limits^{ - } = {{\left( {\sum\nolimits_{i = 1}^{M} {\frac{{\left| {S_{i} - L_{i} } \right|}}{{L_{i} }}} } \right)} \mathord{\left/ {\vphantom {{\left( {\sum\nolimits_{i = 1}^{M} {\frac{{\left| {S_{i} - L_{i} } \right|}}{{L_{i} }}} } \right)} M}} \right. \kern-\nulldelimiterspace} M}$$

where *M* represents the number of roughness parameters, *S* is the measured roughness parameters, and *L* is the values of deduced curves. The results show that the deduced curves are almost identical to the measured data and the average relative errors between the two types of values range from a minimum of 3% to a maximum of 8%.

## Application and discussion

### Actual joint surface measurements

To verify the anisotropic variation behaviour of actual joint surfaces, we first need to collect natural joints. After field investigations, there are three open-pit mines with different lithology (sandstone, tuff, and limestone) in Shaoxing City, Zhejiang Province, China, and the exposed joints are well preserved (see Supplementary Fig. S4 online). Then we select a set of 1000 mm × 1000 mm joints from each mine as the research object and use a portable laser scanner (MetraSCAN 3D, Creaform, Canada) with the scanning accuracy of 0.5 mm to obtain the 3D morphological information of its surface. Finally, three digitized surfaces labeled as S1, T1 and L1 are obtained, which are shown in Supplementary Fig. S5 online.

### Description and analysis of anisotropy

The original anisotropic behaviours are obtained by using the 3D parameter ${{\theta_{\max }^{ * } } \mathord{\left/ {\vphantom {{\theta_{\max }^{ * } } {\left( {C + 1} \right)}}} \right. \kern-\nulldelimiterspace} {\left( {C + 1} \right)}}$. However, due to the complicated distribution in actual joint surfaces, it is difficult to distinguish the locations of the orthogonal directions. To this end, we have developed an adaptive algorithm that can automatically match the roughness parameters of the orthogonal direction for anisotropic analysis. It mainly implements by using the anisotropic analytic function to obtain the deduced curves in each vertical direction on the joint surface, and then compares with the measured data. Among them, the vertical direction with minimum relative error is the location of orthogonal directions. This algorithm is applied to the above three joints, and the calculation results listed in Table 3 indicates that there are obvious orthogonal distribution characteristics in the S1, T1 and L1 joints. In addition, the relative errors of actual anisotropic distribution and analytical anisotropic distribution stable within 10%. Therefore, the anisotropic analytic function can effectively reflect the anisotropic variation behaviour of rock joints.

**Table 3:** Anisotropy analysis of actual joint surfaces.

Joint	Anisotropic distribution	Average relative error (%)	Inferior direction	Advantageous direction	AVC
S1		3.4	${10}0^{ \circ }{-} {28}0^{ \circ }$	${1}0^{ \circ }{-} {19}0^{ \circ }$	0.77
T1		10.7	${14}0^{ \circ }{-} {32}0^{ \circ }$	${5}0^{ \circ }{-} {23}0^{ \circ }$	0.63
L1		7.0	${17}0^{ \circ }{-} {35}0^{ \circ }$	${8}0^{ \circ }{-} {26}0^{ \circ }$	0.98

### Extraction and statistics of index AVC

From the anisotropy distribution of actual joints, the orthogonal direction is not specified in the directions of $0^{ \circ }{-} 180^{ \circ }$ and ${9}0^{ \circ }{-} {27}0^{ \circ }$. To better distinguish the orthogonal direction, the direction with smaller weight of roughness is determined as the inferior direction, and the forward and reverse roughness parameters in this direction are defined as $P_{{I{\text{nf}}}}^{F}$ and $P_{{I{\text{nf}}}}^{R}$, respectively. On the contrary, the direction with greater weight of roughness is determined as the advantageous direction, and the forward and reverse roughness parameters in this direction are defined as $P_{{A{\text{dv}}}}^{F}$ and $P_{{A{\text{dv}}}}^{R}$, respectively. Finally, the *AVC* can be further expressed as follows:

14 $$AVC\,=\,\frac{{P_{{I{\text{nf}}}}^{F} + P_{{I{\text{nf}}}}^{R} }}{{P_{Adv}^{F} + P_{Adv}^{R} }},$$

Therefore, we calculate the AVC of each joint according to the above formula. The results show that the *AVC* values of S1, T1 and L1 joints are 0.77, 0.63 and 0.98, respectively. Compared with the classification of anisotropic variation levels (Table 2), the S1 joint is presents weak anisotropic variation, the T1 joint presents medium anisotropic variation, and the L1 joint is close to isotropic distribution.

## Conclusions

The method for superimposing regular surfaces in orthogonal directions is used to construct the different undulating morphology of the joint surfaces and to characterize the anisotropic behaviour, with index *AVC* being proposed to quantify the degree of anisotropic variation. Based on the assumption of anisotropic arc distribution, the anisotropic distribution function is derived and the capability of the anisotropic variation behaviour are also validated. Some concluding remarks are presented below:

A specific distribution law is investigated in the anisotropy of the joint surface. Such a law shows that a rock joint surface can be simplified to the superposition of regular surfaces in orthogonal directions, and the anisotropy is described by the contribution ratios in these two directions. Six joint surfaces with different contribution ratios are constructed base on the proposed construction method of joint surfaces. The behaviours of anisotropy expressed by 2D and 3D parameters indicate that the contribution ratio can effectively reflect the process of joint surfaces from anisotropy to isotropy.

The *AVC* is proposed by defining the roughness weight ratio in the orthogonal direction of joint surfaces. According to the size of *AVC*, we divide the level of anisotropic variation into five different intervals to describe the degree of anisotropy. Additionally, we derive an anisotropic analytic function based on the assumption of anisotropic arc distribution, and verify it can be used as an effective method for analyzing anisotropic characteristics.

By measuring the anisotropy of three natural rock joints, it is found that there are obvious orthogonal distribution characteristics. The anisotropic analytic function is used to determine the locations of orthogonal directions. The results show that the inferior directions of S1, T1 and L1 joint are ${10}0^{ \circ }{-} 2{8}0^{ \circ }$, ${14}0^{ \circ }{-} {32}0^{ \circ }$ and ${17}0^{ \circ }{-} {35}0^{ \circ }$, and their advantageous directions are ${1}0^{ \circ }{-} {19}0^{ \circ }$, ${5}0^{ \circ }{-} {23}0^{ \circ }$ and ${8}0^{ \circ }{-} {26}0^{ \circ }$, respectively. According to the extracted *AVC* value of each joint, the S1 joint is presents weak anisotropic variation, the T1 joint presents medium anisotropic variation, and the L1 joint is close to isotropic distribution.

In summary, this study provides a new method for accurately judging the strength and weakness of joint mechanical properties and a new index to quantify the degree of anisotropy, which can be used to evaluate the stability of engineering rock mass. However, it is worth noting that *AVC* is proposed based on the anisotropic orthogonal distribution reflected by mathematical statistics method. Whether the other two methods (empirical value method and fractal dimension method) have the same distribution law and then apply AVC to determine the anisotropic variation behavior needs to be further verified. Additionally, the anisotropic distribution function is derived based on a circular curve equation and thus it should be further studied with an elliptical curve. Furthermore, the scale effect of *AVC* will be studied in the future.

## Supplementary information

Supplementary Information.

## References

[1] Barton N, Quadros E (2015). Anisotropy is everywhere, to see, to measure, and to model. Rock Mech. Rock Eng..

[2] Kim H, Cho JW, Song I, Min KB (2012). Anisotropy of elasticmoduli, P-wave velocities, and thermal conductivities of Asan Gneiss, Boryeong Shale, and Yeoncheon Schist in Korea. Eng. Geol..

[3] Li B, Jiang Y, Mizokami T (2014). Anisotropic shear behavior of closely jointed rock masses. Int. J. Rock Mech. Min. Sci..

[4] Jiang Q, Feng XT, Hatzor YH, Hao XJ, Li SJ (2014). Mechanical anisotropy of columnar jointed basalts: An example from the Baihetan hydropower station, China. Eng. Geol..

[5] Dinh QD, Heinz K, Martin H (2013). Brazilian tensile strength tests on some anisotropic rocks. Int. J. Rock Mech. Min. Sci..

[6] Chen GJ (2014). Thermal impact on damaged boom clay and opalinus CLAY: Permeameter and isostatic tests with μCT scanning. Rock Mech. Rock Eng..

[7] Khanlari GR, Heidari M, Sepahigero AA, Fereidooni D (2014). Quantification of strength anisotropy of metamorphic rocks of the Hamedan province, Iran, as determined from cylindrical punch, point load and Brazilian tests. Eng. Geol..

[8] Wang P, Ren F, Miao S (2017). Evaluation of the anisotropy and directionality of a jointed rock mass under numerical direct shear tests. Eng. Geol..

[9] Aydan Ö, Shimizu Y, Kawamoto T (1996). The anisotropy of surface morphology characteristics of rock discontinuities. Rock Mech. Rock Eng..

[10] Grasselli G, Egger P (2003). Constitutive law for the shear strength of rock joints based on three-dimensional surface parameters. Int. J. Rock Mech. Min. Sci..

[11] Baker BR, Gessner K, Holden EJ (2008). Automatic detection of anisotropic features on rock surfaces. Geosphere..

[12] Ge Y, Kulatilake PHSW, Tang H (2014). Investigation of natural rock joint roughness. Comput. Geotech..

[13] Tatone BSA, Grasselli G (2013). Quantitative measurements of fracture aperture and directional roughness from rock cores. Rock Mech. Rock Eng..

[14] Yang ZY, Lo SC (1997). An index for describing the anisotropy of joint surfaces. Int. J. Rock Mech. Min. Sci..

[15] Kulatilake PHSW, Balasingam P, Park J, Morgan R (2006). Natural rock joint roughness quantification through fractal techniques. Geotech. Geol. Eng..

[16] Tatone BSA, Grasselli G (2010). A new 2D discontinuity roughness parameter and its correlation with JRC. Int. J. Rock Mech. Min. Sci..

[17] Babanouri N, Nasab SK, Sarafrazi S (2013). A hybrid particle swarm optimization and multi-layer perceptron algorithm for bivariate fractal analysis of rock fractures roughness. Int. J. Rock Mech. Min. Sci..

[18] Chen S, Zhu W, Yu Q (2015). Characterization of anisotropy of joint surface roughness and aperture by variogram approach based on digital image processing technique. Rock Mech. Rock Eng..

[19] Fathi A, Moradian Z, Rivard P (2015). Geometric effect of asperities on shear mechanism of rock joints. Rock Mech. Rock Eng..

[20] Zhao L, Zhang S, Huang D (2018). Quantitative characterization of joint roughness based on semivariogram parameters. Int. J. Rock Mech. Min. Sci..

[21] Belem T, Homand F, Souley M (2000). Quantitative parameters for rock joint surface roughness. Rock Mech. Rock Eng..

[22] Belem T, Souley M, Homand F (2009). Method for quantification of wear of sheared joint walls based on surface morphology. Rock Mech. Rock Eng..

[23] Belem T (2016). Quantitative parameters of primary roughness for describing the morphology of surface discontinuities at various scales. Geomech. Eng..

[24] Grasselli G (2006). Manuel Rocha medal recipient shear strength of rock joints based on quantified surface description. Rock Mech. Rock Eng..

[25] Sharifzadeh M, Mitani Y, Esaki T (2008). Rock joint surfaces measurement and analysis of aperture distribution under different normal and shear loading using GIS. Rock Mech. Rock Eng..

[26] Tatone, BSA. Quantitative characterization of natural rock discontinuity roughness in-situ and in the laboratory. Masters thesis, Department of Civil Engineering. Toronto, University of Toronto, Canada (2009).

[27] Ge Y, Tang H, Eldin M (2015). A description for rock joint roughness based on terrestrial laser scanner and image analysis. Sci. Rep..

[28] Yang ZY, Taghichian A, Li WC (2010). Effect of asperity order on the shear response of three-dimensional joints by focusing on damage area. Int. J. Rock Mech. Min. Sci..

[29] Tse R, Cruden DM (1979). Estimating joint roughness coefficients. Int. J. Rock Mech. Min. Sci. Geomech. Abstracts..

[30] Huang, M., Hong, C., Du, S. & Luo, Z. Experimental technology for the shear strength of the series-scale rock joint model. *Rock Mech. Rock Eng.* (2020).

