# Supplementary material for: Characterization of rock joint surface anisotropy considering the contribution ratios of undulations in different directions
Source: Sci Rep. 2020 Oct 13;10:17117. doi: 10.1038/s41598-020-74229-z (PMC7555549; doi:10.1038/s41598-020-74229-z)
Supplement: Supplementary file 1 — Supplementary Information. [file 41598_2020_74229_MOESM1_ESM.pdf]

Supplementary Information

Title: Characterization of Rock Joint Surface Anisotropy Considering the Contribution Ratios of Undulations in Different Directions

Authors: Man Huang[a*], Chenjie Hong[a], Chengrong Ma[a], Zhanyou Luo[b], Shigui Du[a]

[a]*Department of Civil Engineering, Shaoxing University, 508 Huancheng West Road, Shaoxing 312000, Zhejiang, China*

[b]*Geotechnical Engineering Institute, Zhejiang University of Science and Technology, 318 Liuhe Road, Hangzhou 310023, Zhejiang, China*

* Corresponding author: Man Huang

Tel.: +86 13615752267

E-mail address: hmcadx@126.com

Table Caption

Table S1 Summary of roughness parameters for anisotropic description.

Table S2 Investigation on the distribution of anisotropy in joint surfaces.

Table S1 Summary of roughness parameters for anisotropic description

Parameter		Dimension	Description	Mathematical expression	References
Directional roughness parameters	$\frac{\theta^*_{max}}{C+1}$	3D	The distribution of the apparent dip (in any direction) of each triangular facet of a triangular irregular network (TIN) surface.	$A_{\theta^*} = A_0\left(\frac{\theta^*_{max} - \theta^*}{\theta^*_{max}}\right)^c$	Tatone 2009
		2D	The distribution of the inclination (in the forward or reverse direction) of each line segment of a 2D roughness profile.	$L_{\theta^*} = L_0\left(\frac{\theta^*_{max} - \theta^*}{\theta^*_{max}}\right)^c$	Tatone and Grasselli 2010
Non-directional roughness parameters	Z_2	2D	The root mean square of the first derivative of the profile height.	$Z_2 = \left[\frac{1}{L}\int_{x=0}^{x=L}\left(\frac{dy}{dx}\right)^2 dx\right]^{1/2}$	Tse and Cruden 1979
	R_p	2D	The ratio of the true length of a 2D roughness profile to its nominal length.	$R_p = \frac{L_t}{L_n} = \frac{\sum_{i=1}^{N-1}\sqrt{(x_{i+1}-x_i)^2+(y_{i+1}-y_i)^2}}{\sum_{i=1}^{N-1}(x_{i+1}-x_i)}$	Maers et al. 1990
	$K_v \times D_{r1d}$	3D	The product of the mean value of fractal dimension and a scale-dependent fractal parameter.	$D_{r1d} = 2 - H$ $2\gamma(x,h)_{h\to 0} = K_v h^{2H}$ $2\gamma(x,h) = \frac{1}{M}\sum_{i=1}^{M}\left[Z(x_i) - Z(x_i+h)\right]^2$	Kulatilake et al. 2006
	SR_v	2D	The ratio of a sill to its range.	$SR_v = \sqrt{2C}/a$	Chen et al. 2015

Table S2 Investigation on the distribution of anisotropy in joint surfaces

Parameter	Dimension	Anisotropic polar map	The angle between the dominant direction and the disadvantaged direction	References
$K_v \times D_{r1d}$	2D	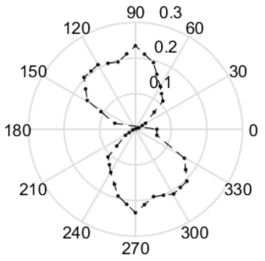	85°	Kulatilake et al., 2006
$\frac{\theta^*_{\max}}{C}$	3D	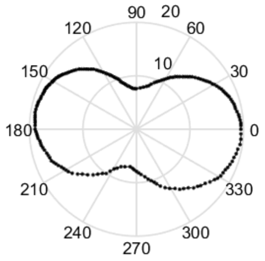	90°	Grasselli et al., 2003
Z_2	2D	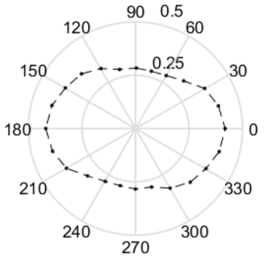	90°	Fathi et al., 2015
$\frac{\theta^*_{\max}}{C+1}$	3D	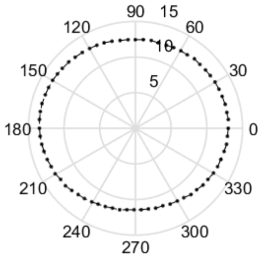	90°	Tatone and Grasselli, 2010

Figure Caption

Fig. S1 Normalized anisotropic distribution map of roughness parameters.

Fig. S2 The indication of the different analysis directions.

Fig. S3 Analytical model.

Fig. S4 Natural rock joints: (a) sandstone; (b) tuff; (c) limestone.

Fig. S5 Three-dimensional morphology of three joints. (a) S1; (b) T1; (c) L1.

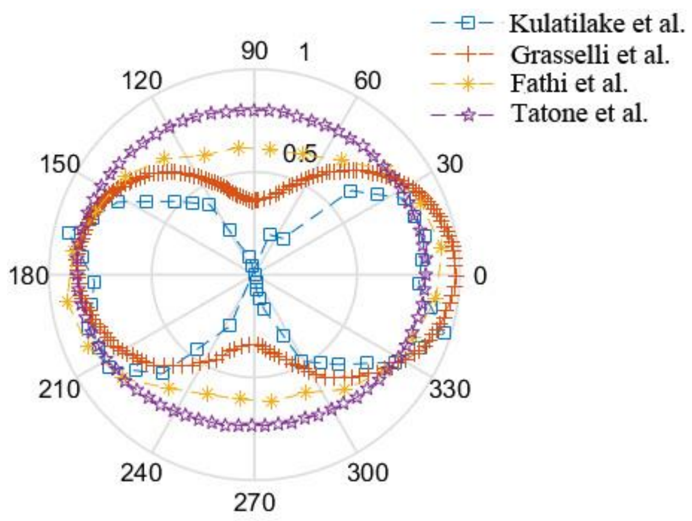

Fig. S1 Normalized anisotropic distribution map of roughness parameters

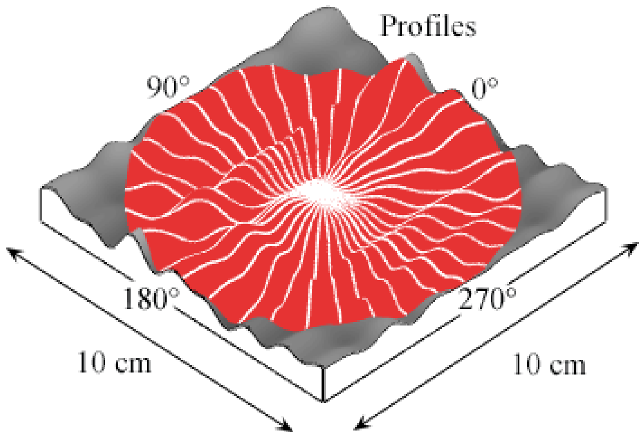

Fig. S2 The indication of the different analysis directions

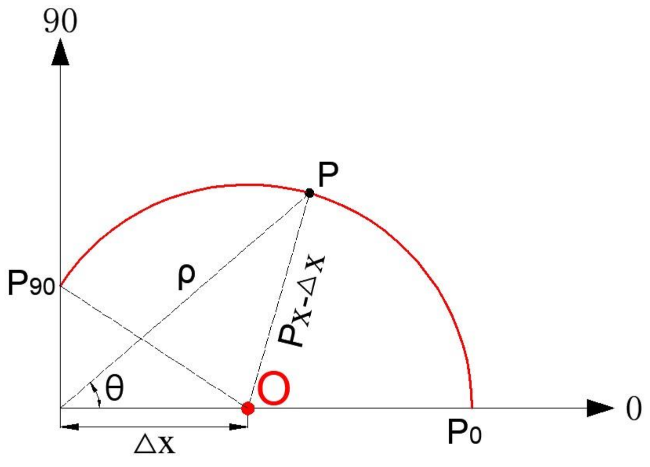

Fig. S3 Analytical model

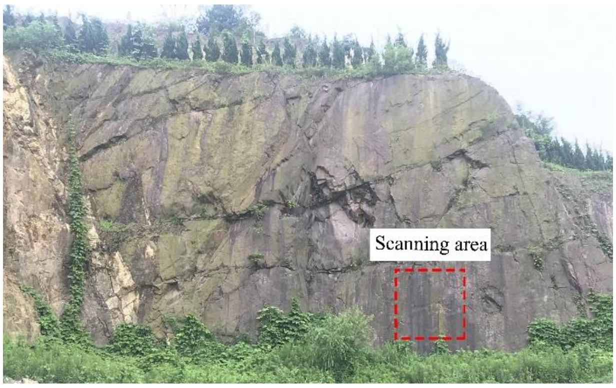

(a)

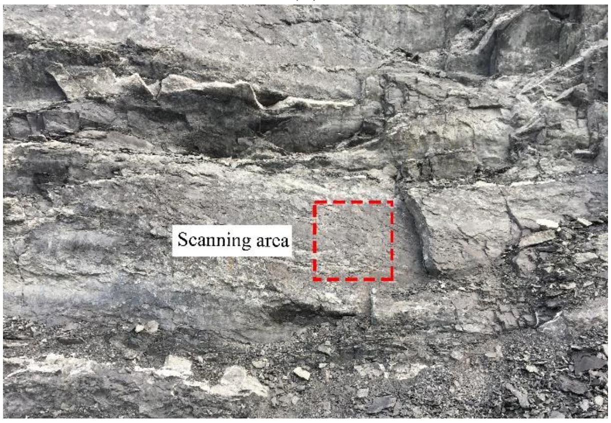

(b)

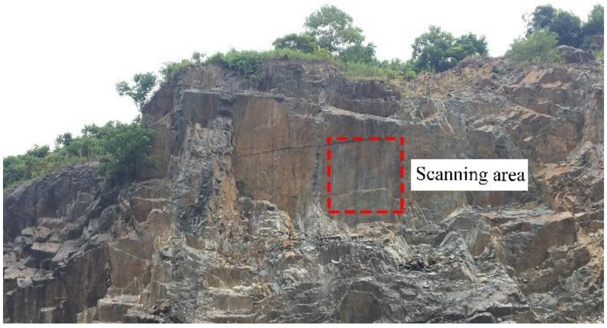

(c)

Fig. S4 Natural rock joints: (a) sandstone; (b) tuff; (c) limestone

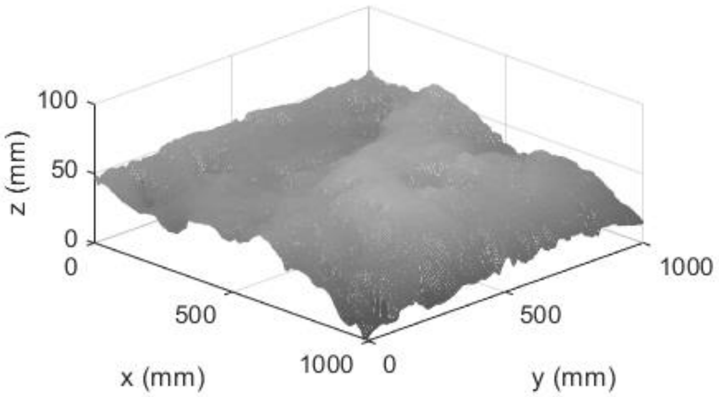

(a)

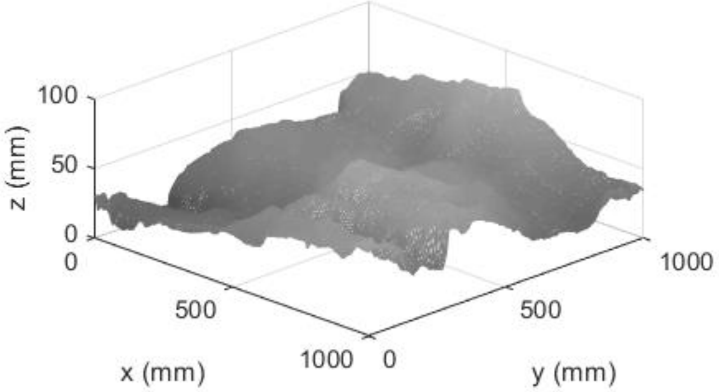

(b)

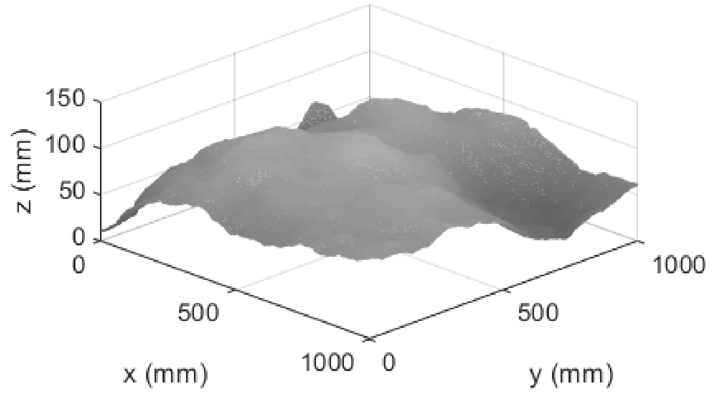

(c)

Fig. S5 Three-dimensional morphology of three joints. (a) S1; (b) T1; (c) L1
